# Independent Dose–Response Associations between Fetuin-A and Lean Nonalcoholic Fatty Liver Disease

**DOI:** 10.3390/nu13092928

**Published:** 2021-08-24

**Authors:** Chia-Wen Lu, Yi-Chen Lee, Chien-Hsieh Chiang, Hao-Hsiang Chang, Wei-Shiung Yang, Kuo-Chin Huang

**Affiliations:** 1 Graduate Institute of Clinical Medicine, College of Medicine, National Taiwan University, Taipei 100, Taiwan; biopsycosocial@gmail.com (C.-W.L.); wsyang@ntu.edu.tw (W.-S.Y.); 2 Department of Family Medicine, National Taiwan University Hospital, Taipei 100, Taiwan; jiansie@ntu.edu.tw (C.-H.C.); allanchanghs@gmail.com (H.-H.C.); 3 Department of Family Medicine, College of Medicine, National Taiwan University, Taipei 100, Taiwan; 4 Department of Family Medicine, National Taiwan University Hospital Bei-Hu Branch, Taipei 108, Taiwan; msar224@gmail.com; 5 Genome and Systems Biology Degree Program, Academia Sinica and National Taiwan University, Taipei 100, Taiwan; 6 Department of Internal Medicine, National Taiwan University Hospital, Taipei 100, Taiwan

**Keywords:** central obesity, fetuin-A, lean NAFLD, insulin resistance

## Abstract

Patients with lean NAFLD make up an increasing subset of liver disease patients. The association between lean NAFLD and feutin-A, which serves as a hepatokine and adipokine, has never been examined. Our study aimed to explore the association of serum fetuin-A among lean and non-lean patients. The study comprised 606 adults from the community, stratified into lean or non-lean (BMI </≥ 24 kg/m^2^) and NAFLD or non-NAFLD (scoring of ultrasonographic fatty liver indicator, US-FLI ≥ 2/< 2). Multivariate logistic regression analyses were performed to estimate the odds ratio of having NAFLD among the tertiles of fetuin-A after adjustment. The least square means were computed by general linear models to estimate marginal means of the serum fetuin-A concentrations in relation to the NAFLD groups. The odds ratio (OR) of having NAFLD for the highest versus the lowest tertile of fetuin-A was 2.62 (95% CI: 1.72–3.98; *p* for trend < 0.001). Stratifying by BMI, the OR of having lean NAFLD for the highest versus the lowest tertile of fetuin-A was 2.09 (95% CI: 1.09–3.98; *p* for trend 0.026), while non-lean NAFLD had no significant association with the fetuin-A gradient after adjustments. Fetuin-A was positively associated with lean NAFLD after adjusting for central obesity and insulin resistance.

## 1. Introduction

Nonalcoholic fatty liver disease (NAFLD) is a growing health concern due to its increasing incidence and prevalence and its impact on associated comorbidities. The incidence of NAFLD is 28–52 per 1000 person-years, and the prevalence of NAFLD is approximately 25% [1]. It is well established that NAFLD is commonly associated with obesity, type 2 diabetes (T2DM), dyslipidemia, and metabolic syndrome (MetS) [2]. Therefore, a synonymous terminology is developing for diseases ranging from NAFLD to metabolic-associated fatty liver disease (MAFLD) [3]. However, there has been an increasing subset of patients with lean NAFLD, where they have NAFLD but also a normal body mass index [4]. Compared with non-lean NAFLD, patients with lean NAFLD are younger and have higher hemoglobin levels [5], an elevated ALT/AST ratio [6], and less insulin resistance and MetS [7]. Compared with healthy subjects, lean NAFLD patients have more dyslipidemia [8] and easier central obesity and insulin resistance [9]. Overall, in terms of phenotype, patients with non-lean NAFLD share metabolic features of insulin resistance and dyslipidemia with lean NAFLD patients [7]. From a histological perspective, lean NAFLD seems to have less severe steatosis [10], where >5% of hepatocytes are considered abnormal; lean NAFLD also has similar prevalence of NASH, where ballooning degeneration, lobular or portal inflammation, and fibrosis are present [10,11]. In general, the limited data, conflicting results, and increasing population of lean NAFLD patients have evoked remarkable concern.

Fetuin-A, also named Alpha2-Heremans-Schmid glycoprotein, is synthesized in hepatocytes and secreted into the bloodstream [12]. One of the most documented functions of fetuin-A is to act as an endogenous inhibitor of the insulin receptor tyrosine kinase, which triggers insulin resistance [13]. Therefore, fetuin-A has been highly correlated with T2DM, obesity, and MetS in previous studies [14,15]. Recently, fetuin-A was assumed to act as an endogenous ligand of Toll-like receptor 4 to stimulate chronic adipose inflammation [16]. Fetuin-A stimulates the secretion of inflammatory cytokines, such as TNF-alpha and interleukin-6, in adipose tissue [17]. With roles in both insulin resistance and chronic inflammation, circulating fetuin-A levels have been found to be significantly correlated with NAFLD patients [18]. However, the association between lean NAFLD and fetuin-A has never been studied. Therefore, we focused on a young adult population and conducted a community-based investigation to examine the clinical characteristics and metabolic factors of four groups: lean (+) NAFLD (−), lean (+) NAFLD (+), lean (−) NAFLD (−), and lean (−) NAFLD (+). The study also aimed to explore the association of serum gradients of fetuin-A among four groups (lean/NAFLD: +/−, +/+, −/−, −/+) after adjusting for insulin resistance and central obesity.

## 2. Materials and Methods

### 2.1. Study Subjects

This study was conducted in the community of Hsinchu City, Northern Taiwan. All the participants completed standardized questionnaires through individual interviews. The exclusion criteria were excessive alcohol use, which was defined as drinking more than 20 g of alcohol daily for women and 30 g for men, and chronic liver diseases, which included chronic hepatitis, autoimmune, drug-induced, vascular, and inherited hemochromatosis, as well as Wilson disease. According to the recommendation of World Health Organization, both men and women were suggested to drink less than two standard drinks per day, i.e., 20 g of pure ethanol per day [19]. The amount of alcohol in any drink is calculated by the following equation: pure alcohol mass equals volume (L) × alcohol percentage (%) × volumetric mass density (g/L) [20]. Subjects who drank more than the limited amount were excluded to confirm that we only enrolled NAFLD. In total, 606 adults aged 20 to 80 years old were enrolled. Information about age, gender, personal habits including cigarette smoking and exercise habits, and previous diseases was obtained after informed consent forms were signed. Current smokers were defined as those who had been smoking for more than 6 months prior to participating in this study. Noncurrent smokers were defined as those who had quit smoking for more than 12 months before the study or who had never been smokers. Exercise habit was defined by the following yes or no question: “Do you have a regular exercise habit?”. Weight and height were measured by a standard electronic scale and stadiometer. Waist circumference (WC) was measured at the level of the umbilicus by a by the same trained operator while the nearest millimeter was recorded. Blood pressure (BP) was measured by a sphygmomanometer. The first and fifth Korotkoff phases were used to determine systolic blood pressure (BP) and diastolic BP, respectively [21] This study was approved by the Institutional Review Board of National Taiwan University Hospital (IRB NO. 201210012RIC).

### 2.2. Ultrasonography Assessment

Abdominal ultrasonography was performed after at least eight hours of fasting by a well-trained examiner with a 3.5–5 MHz transducer and a high-resolution B-mode scanner (Hitachi Aloka ProSound α 6). The ultrasound measurements were performed by three experienced research physicians. Before the study, all three physicians reached a consensus regarding the standard procedure for ultrasound scanning, including the scoring of ultrasonographic fatty liver indicator (US-FLI) and the sequence of acquiring liver images. The severity of NAFLD was calculated using the US-FLI score, which ranges from 0 to 8 [22]. The US-FLI is composed of five indicators: (1) the presence of liver-kidney contrast graded as mild/moderate (score 2) and severe (score 3); and (2) the presence (score 1) or absence (score 0) of posterior attenuation of the ultrasound beam, vessel blurring, difficult visualization of the gallbladder wall, difficult visualization of the diaphragm, and areas of focal sparing (score of 1 each). The subjects were then divided into four groups: (1) lean non-NAFLD group: US-FLI score < 2, BMI < 24 kg/m^2^; (2) lean NAFLD group: US-FLI score ≥ 2, BMI< 24 kg/m^2^; (3) non-lean, non-NAFLD group: US-FLI score < 2, BMI ≥ 24 kg/m^2^; (4) non-lean NAFLD group: US-FLI score ≥ 2, BMI ≥ 24 kg/m^2^.

### 2.3. Blood Analysis

Venous blood was sampled after ≥8 h of fasting. Serum glucose, total cholesterol, high-density lipoprotein cholesterol (HDL-C), low-density lipoprotein cholesterol (LDL-C), and triglycerides were measured by an automatic spectrophotometric assay (HITACHI 7250, Tokyo, Japan). Fasting insulin levels were examined by a microparticle enzyme immunoassay using an AxSYM system (Abbott Laboratories, Dainabot Co., Tokyo, Japan). We estimated the intensity of insulin resistance by an indirect assessment, the homeostatic model assessment of insulin resistance (HOMA-IR). The convert equation was HOMA-IR = fasting insulin × fasting plasma glucose/22.5, with glucose shown in mmol/L and insulin shown in mU/L [23]. Serum fetuin-A was measured using a quantitative sandwich enzyme immunoassay technique after a 4000-fold dilution. This immunoassay was calibrated against highly purified NS0-expressing recombinant human fetuin-A (R&D Inc. Minneapolis, MN, USA).

### 2.4. Statistical Analysis

Subjects were sorted into tertiles according to the serum levels of fetuin-A. Basic demographic characteristics are shown as the mean ± standard deviation for the continuous parameters and cases (%) for the categorical parameters. Multivariate logistic regression analyses were performed to calculate the odds ratio of having NAFLD among the tertiles of fetuin-A after adjustment for age, gender, personal habits, WC, and the HOMA-IR, stratified by BMI or not. The least square means were computed by general linear models to estimate marginal means of the serum fetuin-A concentrations in relation to the NAFLD groups after adjusting for age, gender, personal habits, weight circumference, and the homeostasis model assessment of insulin resistance. We conducted statistical analyses by applying SPSS statistical software (V.17, SPSS, Chicago, IL, USA). We assumed a statistical significance whenever the *p* value < 0.05.

## 3. Results

### 3.1. General Characteristics

The basic characteristics of the participants are shown in Table 1. The mean age of the participants was 42.6 ± 11.5 years old, the median was 41.0 years old (25th/75th: 34.0/50.0 years old), and 61.7% of the participants were female and 38.3% of the participants were male. The mean serum concentrations of fetuin-A were 689.4 ± 672.4 mg/L, 882.6 ± 731.3 mg/L, 829.3 ± 429.3 mg/L, and 855.9 ± 467.0 mg/L in the four groups, respectively. The scattered plots and box plot representing the distribution of subjects among four groups are shown in Supplementary Figure S1. The highest level of fetuin-A was found in the lean NAFLD group. In a post hoc analysis (Table 2), the lean NAFLD group shared similar metabolic factors with the non-lean, non-NAFLD group. However, patients in the former group had a presentation of NAFLD and patients in the latter had a significantly higher BMI, waist circumference, and body fat percentage. Both lean and non-lean NAFLD had high levels of fetuin-A, while non-lean NAFLD apparently had more metabolic factors and high BMI, waist circumference, and body fat percentage. This section may be divided by subheadings. It should provide a concise and precise description of the experimental results, their interpretation, as well as the experimental conclusions that can be drawn.

### 3.2. Association of Fetuin-A and NAFLD

To further clarify the association between the concentration gradients of fetuin-A and NAFLD, multiple logistic regression analyses were applied to examine the odds ratios (ORs) of having NAFLD derived from tertiles of serum fetuin-A levels in Table 3. The OR of having NAFLD for the highest versus the lowest tertile of fetuin-A was 2.62 (95% CI: 1.72–3.98; *p* for trend < 0.001) adjusting for age, gender, and personal habits. After adjustment for age, gender, personal habits, and WC, the OR of having NAFLD for the highest versus the lowest tertile of fetuin-A was 1.80 (95% CI: 1.10–2.94, *p* for trend 0.02). However, after further adjusting for the HOMA-IR, the ORs became insignificant (1.5; 95% CI: 0.92–2.67; *p* for trend 0.099).

Stratified by BMI, the ORs of having NAFLD derived from multiple logistic regression analyses in tertiles of serum fetuin-A are shown in Table 4. When BMI < 24 kg/m^2^, the crude OR of having NAFLD for the highest versus the lowest tertile of fetuin-A was 1.95 (95% CI: 1.14–3.34; *p* for trend < 0.018). After adjusting for age, gender, personal habits, WC, and the HOMA-IR, the OR of having NAFLD for the highest versus the lowest tertile of fetuin-A was 2.09 (95% CI: 1.09–3.98; *p* for trend 0.026). When BMI > 24 kg/m^2^, both the crude ORs and the adjusted ORs of having NAFLD for the highest versus the lowest tertile of fetuin-A were insignificant, being 1.35 (95% CI: 0.57–3.21; *p* for trend < 0.603) and 0.69 (95% CI: 0.24–1.95; *p* for trend 0.422), respectively.

**Figure 1:**
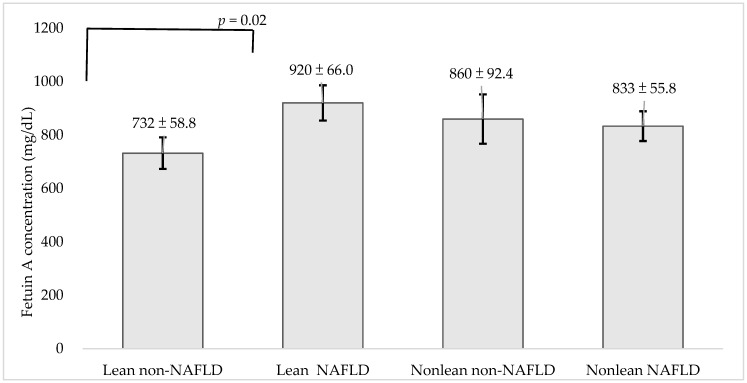
Comparison of serum concentrations of fetuin-A in relation to the groups of NAFLD after adjusting age, gender, personal habits, weight circumference, and the homeostasis model assessment of insulin resistance by least square means method. Data are shown as mean ± SD with error bars. Statistical significance was only found between lean non-NAFLD and lean NAFLD (*p* < 0.05) groups, but not found between the non-lean non-NAFLD and non-lean NAFLD (*p* = 0.798) groups.

The least square means (±SDs) of the serum fetuin-A concentrations in relation to the four groups were 732.4 (617.0–847.9) mg/L, 920.3 (790.5–1050.1) mg/L, 860.0 (678.5–1041.6) mg/L, and 833.3 (723.7–942.9) mg/L, respectively, after adjusting for age, gender, personal habits, WC, and the HOMA-IR (Figure 1).

## 4. Discussion

This is the first study to demonstrate that there is a positive association between the serum fetuin-A gradient and the risk of lean NAFLD. First, a 2.09-fold risk of lean NAFLD was found in the highest tertile compared with the lowest tertile of serum fetuin-A, while no significance was found in non-lean NAFLD. Second, we also found that there was a dose–response relationship between the serum fetuin-A gradient and non-lean NAFLD after adjusting for age, gender, personal habits, WC, and the HOMA-IR (*p* for trend < 0.05). Third, both lean and non-lean NAFLD had high levels of fetuin-A, while non-lean NAFLD apparently had more metabolic factors and higher BMI, waist circumference, and body fat percentage. The persistence of a direct relationship between fetuin-A and the risk of lean NAFLD after adjusting for WC and the HOMA-IR implied that still unidentified factors affected this association beyond the central obesity and insulin resistance that were only captured in the lean subjects.

The name fetuin implies that its amount is highest in fetal blood. Fetuin is found in significantly lower concentrations in adults [24] and serves pleiotropic functions. In adults, fetuin-A is secreted by hepatocytes and adipocytes and predominantly (>95%) expressed in the liver [25]. It is well known that fetuin-A is involved in the development of insulin resistance in both animal and human studies [26,27], and thus contributes to the development of NAFLD. Fetuin-A promotes lipid-induced inflammation by binding free fatty acids to Toll-like receptor 4 in animal studies [16,28], most likely contributing even further to the progression of NAFLD. It is not surprising that fetuin-A levels were significantly elevated in NAFLD patients in previous studies [18]. In biopsy-proven human studies, both circulating levels of fetuin-A and the hepatic expression of fetuin-A were higher in NAFLD patients than in healthy controls regardless of the histological state and BMI class [29], implying that the BMI-oriented concept for NAFLD or MAFLD might need to be reconsidered. To date, there have been no data on the relationship or the underlying mechanisms between lean NAFLD and the serum gradient of fetuin-A. We boldly hypothesize that, although lean NAFLD is associated with fewer metabolic dysfunctions than non-lean NAFLD, it might be prone to more progressive inflammation and oxidative stress. Experimental studies have shown that fetuin-A promotes the expression of proinflammatory cytokines at the mRNA and protein levels [12,30] and chronically responds to inflammatory stimuli [31], leading to the progression from steatohepatitis to NASH [32,33]. In a study cohort comprising 1339 Caucasian biopsy-proven NAFLD patients, it was found that both lean and non-lean NAFLD may progress to advanced liver disease, metabolic comorbidities, cardiovascular disease, and liver-related mortality, independent of the progression to obesity [34].

It is interesting but puzzling that fetuin-A is prone to be elevated in early NAFLD, and that it is more prominent in lean NAFLD. We boldly hypothesized that the amount of adipose composition reflects the capacity of lipid storage to some extent. Therefore, lacking adipose tissue in lean subjects is thought to be because of less fat storage capacity and is associated with lipid accumulation in ectopic sites [35,36]. After triglycerides are eventually saturated in adipocytes, the liver was recognized as the most sensitive and vulnerable ectopic site for fat deposition, leading to fatty liver disease [37,38]. Although lipodystrophy might be specific for acquired or congenital loss of adipose tissue, more and more evidence supported that within lean people in the general population, some features of lipodystrophy exist, i.e., insulin resistance and accumulation of lipids in the liver [39,40]. Furthermore, an animal model has demonstrated that a lean mouse phenotype with fatty liver was probably a consequence of adipocyte dysfunction [41]. Inspiringly, we found that lean NAFLD subjects shared similar risks of metabolic factors, including fasting glucose, insulin resistance, lipid profiles, and blood pressure, with non-lean, non-NAFLD subjects. In our data, lean NAFLD subjects had a normal BMI and fatty liver disease, while non-lean non-NAFLD subjects were overweight or obese with a significantly higher fat percentage and waist circumference. In line with our findings, lipodystrophy limited the lipid accumulation in lean NAFLD subjects, causing ectopic fat accumulation in the liver. We thus inferred that the role of fetuin-A, majorly as a hepatokine and minorly as an adipokine, was reasonable for the highest concentration in the lean NAFLD group.

It has been observed that lean NAFLD patients are younger and have fewer metabolic clinical features but share similar histological severity, comorbidities, and mortality with their non-lean counterparts [42]. Lean NAFLD subjects develop NAFLD prior to obesity and metabolic dysfunction, and conventional metabolic factors cannot be used for early detection. Since liver fat accumulation and chronic inflammation are very sensitive and early indicators in these subsets, fetuin-A, as a hepatokine and an adipokine, could be used as a surrogate biomarker independent of central obesity and insulin resistance. The strengths of our study therefore cannot be ignored. We were the first to link the serum level of fetuin-A with lean NAFLD and to demonstrate a dose escalation of fetuin-A for the risk of lean NAFLD.

There are some limitations in our study. First, this is a cross-sectional study, and we could not interfere with the causal relationship between lean NAFLD and the serum gradient of fetuin-A. Despite the collection and adjustment of probable confounders, there could be unmeasured and undefined factors indicating possible residual effects. For example, the duration of NAFLD may potentially influence serum fetuin-A levels over time, but we did not collect longitudinal data from lean or non-lean NAFLD individuals. The relationship between lean NAFLD and fetuin-A warrants more investigation through basic and clinical studies to clarify the pathophysiology of lean NAFLD and fetuin-A with well-designed animal models and prospective cohorts. Second, we did not perform liver biopsy, which is the gold standard for the diagnosis of NAFLD. Although the ultrasonographic approach could not distinguish the severity of NAFLD, it has been acknowledged as a screening tool for NAFLD [2]. In addition, we applied US-FLI, an extensively applied ultrasonographic scoring system, as a substitute modality for the diagnosis of NAFLD [22,43]. Although the bias of misclassification by ultrasound could exist and attenuate the association, we still demonstrated a statistical significance between the non-NAFLD and NAFLD group. Furthermore, we did not check inflammatory markers, such as TNF-alpha and IL-6 levels, as well as their association with fetuin-A, to clarify the inflammatory status probably related to the underlying mechanism. Further studies should focus on the combination of ultrasonographic assessment and surrogate biomarkers to improve the accuracy and precision of noninvasive approaches for NAFLD.

## 5. Conclusions

In conclusion, we found that serum fetuin-A has a dose–response association with lean NAFLD independent of insulin resistance and central obesity. In order to address the increasing subset of lean NAFLD patients and reappraise BMI-approached MAFLD, further investigations are needed to explore the mechanisms connecting fetuin-A to lean NAFLD as well as their clinical application.

## Figures and Tables

**Table 1:** Baseline characteristics among the lean, non-lean, NAFLD, and non-NAFLD groups.

	Lean		Non-Lean		*p* Value
	Non-NAFLD	NAFLD	Non-NAFLD	NAFLD	
	N = 227	N = 108	N = 54	N = 217	
Age (years)	41.1 ± 11.0	42.6 ± 11.6	44.5 ± 11.3	43.7 ± 11.8	0.061
Male (%)	47 (20.7%)	37 (34.3%)	25 (46.3%)	123 (56.7%)	<0.001
Female (%)	180 (79.3%)	71 (65.7%)	29 (53.7%)	94 (43.3%)	<0.001
BMI (kg/m^2^)	20.6 ± 1.8	21.9 ± 1.5	26.0 ± 1.7	28.1 ± 4.0	<0.001
WC (cm)	73.1 ± 6.1	77.6 ± 6.5	85.4 ± 6.2	91.1 ± 8.3	<0.001
Body fat (%)	25.6 ± 6.2	26.6 ± 6.0	30.0 ± 7.9	32.4 ± 8.4	<0.001
Systolic BP	115.7 ± 15.7	121.6 ± 15.3	122.6 ± 17.0	130.4 ± 15.3	<0.001
Diastolic BP	72.9 ± 11.2	77.2 ± 9.5	77.9 ± 13.8	82.2 ± 12.2	<0.001
TCHO (mmol/L)	190.0 ± 33.8	196.9 ± 39.8	194.6 ± 29.3	201.7 ± 35.5	0.007
TG (mmol/L)	74.2 ± 37.2	109.2 ± 78.9	95.0 ± 43.5	160.2 ± 113.8	<0.001
HDL-C (mmol/L)	66.7 ± 15.0	57.3 ± 13.2	59.5 ± 13.5	49.7 ± 12.6	<0.001
LDL-C (mmol/L)	114.5 ± 31.2	125.4 ± 37.1	123.0 ± 29.2	131.7 ± 32.5	<0.001
Glucose (mmol/L)	83.7 ± 13.0	85.3 ± 8.7	87.0 ± 10.4	94.2 ± 22.8	<0.001
Insulin (U/mL)	5.29 ± 4.24	6.77 ± 5.21	7.1 ± 3.9	11.5 ± 8.9	<0.001
HOMA-IR	0.68 ± 0.55	0.86 ± 0.65	0.91 ± 0.49	1.49 ± 1.10	<0.001
Current smoker (%)	16 (7.0)	11 (10.2)	5 (9.3)	35 (16.1)	0.022
Exercise (%)	100 (44.1)	46 (42.6)	27 (50.0)	92 (42.4)	0.782
GOT	20.3 ± 6.8	21.7 ± 7.0	21.5 ± 5.9	25.8 ± 10.2	<0.001
GPT	17.2 ± 9.4	23.8 ± 16.5	21.4 ± 10.6	36.7 ± 27.8	<0.001
CRP (mg/dL)	0.11 ± 0.31	0.10 ± 0.13	0.17 ± 0.28	0.22 ± 0.25	<0.001
Metabolic factors	0.39 ± 0.62	0.91 ± 0.89	1.15 ± 0.90	2.14 ± 1.18	<0.001
MetS (%)	2 (2.5)	6 (7.5)	4 (5.0)	68 (85)	<0.001
Fetuin-A (mg/L)	689.4 ± 672.4	882.6 ± 731.3	829.3 ± 429.3	855.9 ± 467.0	0.009

ANOVA was applied to test the difference among groups. Abbreviations: NAFLD, nonalcoholic fatty liver disease; BMI, body mass index; WC, waist circumference; BP, blood pressure; TCHO, total cholesterol; TG, triglycerides, HDL-C, high-density lipoprotein cholesterol; LDL-C, low-density lipoprotein cholesterol; HOMA-IR, homeostasis model assessment of insulin resistance; CRP, C-reactive protein; MetS: metabolic syndrome. Current smokers were defined as those who had been smoking for more than 6 months prior to participating in this study. Noncurrent smokers were defined as those who had quit smoking for more than 12 months before the study or who had never been smokers. Exercise habit was defined by the following yes or no question: “Do you have a regular exercise habit?”. Significance level: *p* < 0.05.

**Table 2:** Comparison of lean, non-lean, NAFLD, and non-NAFLD groups in metabolic variables.

Lean/NAFLD:	+/− vs. +/+	+/− vs. −/−	+/− vs. −/+	+/+ vs. −/−	+/+ vs. −/+	−/− vs. −/+
Age (years)	0.663	0.199	0.079	0.754	0.856	0.966
Male (%)	0.059	0.002	<0.001	0.400	<0.001	0.451
BMI (kg/m^2^)	0.001	<0.001	<0.001	<0.001	<0.001	<0.001
WC (cm)	<0.001	<0.001	<0.001	<0.001	<0.001	<0.001
Body fat (%)	0.610	<0.001	<0.001	0.024	<0.001	0.122
Systolic BP	0.007	0.019	<0.001	0.977	<0.001	0.006
Diastolic BP	0.007	0.022	<0.001	0.986	0.002	0.075
TCHO (mmol/L)	0.339	0.822	0.003	0.980	0.658	0.553
TG (mmol/L)	0.001	0.321	<0.001	0.711	<0.001	<0.001
HDL-C (mmol/L)	<0.001	0.003	<0.001	0.745	<0.001	<0.001
LDL-C (mmol/L)	0.023	0.315	<0.001	0.971	0.359	0.296
Glucose (mmol/L)	0.833	0.5448	<0.001	0.928	<0.001	0.024
Insulin (U/mL)	0.303	0.352	<0.001	0.994	<0.001	<0.001
HOMA-IR	0.303	0.324	<0.001	0.990	<0.001	<0.001
GOT	0.499	0.767	<0.001	1.000	<0.001	0.003
GPT	0.017	0.463	<0.001	0.876	<0.001	<0.001
CRP (mg/dL)	0.961	0.439	<0.001	0.321	<0.001	0.672
Fetuin-A (mg/L)	0.030	0.413	0.019	0.951	0.981	0.991

Turkey post hoc analysis was performed to compare each two groups within the four groups to know which two groups were significantly different in the ANOVA analysis. Four groups: lean (+) NAFLD (−), lean (+) NAFLD (+), lean (−) NAFLD (−), and lean (−) NAFLD (+).

**Table 3:** Odds ratios of having NAFLD derived from multiple logistic regression analyses in tertiles of serum fetuin-A levels.

	Q1 (N = 202) (≤821 mg/L)	Q2 (N = 201) (822–1012 mg/L)	Q3 (N = 203) (1013–1224 mg/L)	*p* for Trend
Model 1	1.00	2.49 (1.64–3.77) **	2.62 (1.72–3.98) **	<0.001
Model 2	1.00	1.55 (0.94–2.56)	1.80 (1.10–2.94) *	0.020
Model 3	1.00	1.49 (0.87–2.57)	1.57 (0.92–2.67)	0.099

Model 1: adjustment of age, gender, and personal habits. Model 2: adjustment of age, gender, personal habits, and waist circumference. Model 3: adjustment of age, gender, personal habits, waist circumference, and the homeostasis model assessment of insulin resistance. * For *p* < 0.05; ** For *p* < 0.001.

**Table 4:** Odds ratios of having NAFLD derived from multiple logistic regression analyses in tertiles of serum fetuin-A levels, with stratification by BMI.

**Lean NAFLD**				
	**Q1 (N = 158)**	**Q2 (N = 75)**	**Q3 (N = 102)**	***p* for Trend**
Model 1	1.00	1.01 (0.53–1.90)	1.95 (1.14–3.34) *	0.018
Model 2	1.00	1.26 (0.63–2.50)	2.26 (1.26–4.07) *	0.007
Model 3	1.00	1.33 (0.63–2.82)	2.09 (1.09–3.98) *	0.026
**Overweight/Obese NAFLD**				
	**Q1 (N = 44)**	**Q2 (N = 126)**	**Q3 (N = 101)**	***p* for Trend**
Model 1	1.00	1.48 (0.65–3.38)	1.35 (0.57–3.21)	0.603
Model 2	1.00	1.20 (0.47–3.02)	0.89 (0.34–2.33)	0.688
Model 3	1.00	0.95 (0.35–2.56)	0.69 (0.24–1.95)	0.422

Model 1: adjustment of age, gender, and personal habits. Model 2: adjustment of age, gender, personal habits, and waist circumference. Model 3: adjustment of age, gender, personal habit, waist circumference, and the homeostasis model assessment of insulin resistance. * For *p* < 0.05.

## Supplementary Materials

The following are available online at https://www.mdpi.com/article/10.3390/nu13092928/s1, Figure S1A. Scattered plots of fetuin-A concentration among four groups. The data showed as a collection of points, each having the value of fetuin-A concentration on the vertical axis and the category of group in the horizontal axis. Figure S1B. Box plot of fetuin-A concentration among four groups. The lines from bottom to top represented Q1, Q2, Q3 and Q4 values, respectively, The box showed the interquartile range, the distance between Q3 and Q1. The larger data than Q4 pointed any outliers.
