# Supplementary material for: Independent Dose–Response Associations between Fetuin-A and Lean Nonalcoholic Fatty Liver Disease
Source: Nutrients. 2021 Aug 24;13(9):2928. doi: 10.3390/nu13092928 (PMC8468081; doi:10.3390/nu13092928)
Supplement: Supplementary file 1 [file nutrients-13-02928-s001.zip › nutrients-1306224-supplementary.pdf]

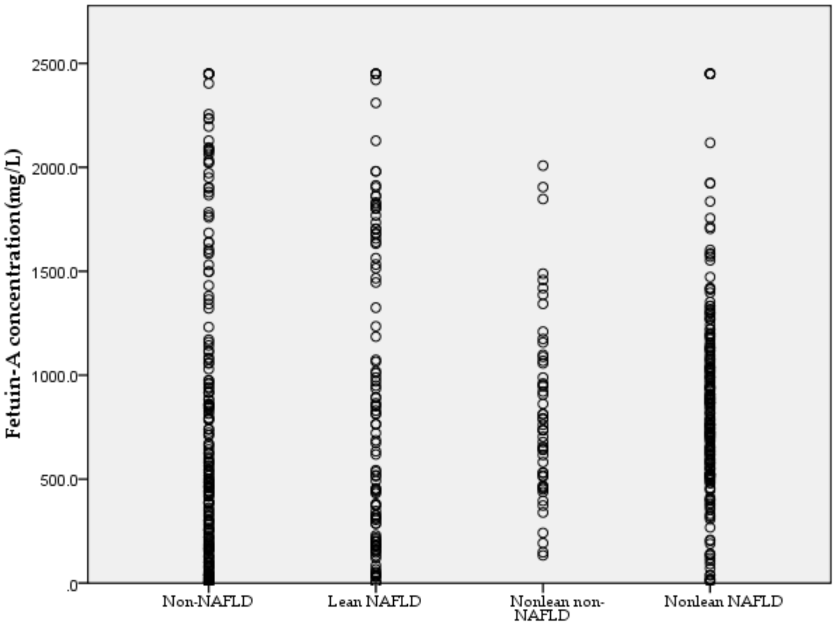

Suppl.1A. Scattered plots of fetuin-A concentration among four groups. The data showed as a collection of points, each having the value of fetuin-A concentration on the vertical axis and the category of group in the horizontal axis.

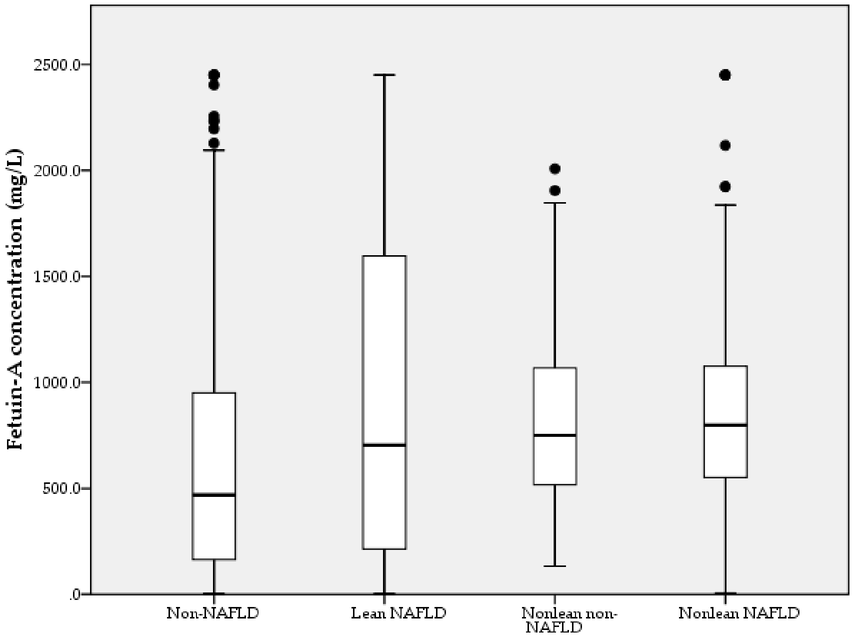

Suppl. 1B. Box plot of fetuin-A concentration among four groups.The lines from bottom to top represented Q1, Q2, Q3 and Q4 values, respectively, The box showed the interquartile range, the distance between Q3 and Q1. The larger data than Q4 pointed any outliers.
